# Defining the Metabolic Functions and Roles in Virulence of the *rpoN1* and *rpoN2* Genes in *Ralstonia solanacearum* GMI1000

**DOI:** 10.1371/journal.pone.0144852

**Published:** 2015-12-11

**Authors:** Benjamin R. Lundgren, Morgan P. Connolly, Pratibha Choudhary, Tiffany S. Brookins-Little, Snigdha Chatterjee, Ramesh Raina, Christopher T. Nomura

**Affiliations:** 1 Department of Chemistry, State University of New York–College of Environmental Science and Forestry, Syracuse, New York, United States of America; 2 Department of Biology, Syracuse University, Syracuse, New York, United States of America; 3 Center for Applied Microbiology, State University of New York–College of Environmental Science and Forestry, Syracuse, New York, United States of America; Gyeongnam National University of Science and Technology, REPUBLIC OF KOREA

## Abstract

The alternative sigma factor RpoN is a unique regulator found among bacteria. It controls numerous processes that range from basic metabolism to more complex functions such as motility and nitrogen fixation. Our current understanding of RpoN function is largely derived from studies on prototypical bacteria such as *Escherichia coli*. *Bacillus subtilis* and *Pseudomonas putida*. Although the extent and necessity of RpoN-dependent functions differ radically between these model organisms, each bacterium depends on a single chromosomal *rpoN* gene to meet the cellular demands of RpoN regulation. The bacterium *Ralstonia solanacearum* is often recognized for being the causative agent of wilt disease in crops, including banana, peanut and potato. However, this plant pathogen is also one of the few bacterial species whose genome possesses dual *rpoN* genes. To determine if the *rpoN* genes in this bacterium are genetically redundant and interchangeable, we constructed and characterized Δ*rpoN1*, Δ*rpoN2* and Δ*rpoN1* Δ*rpoN2* mutants of *R*. *solanacearum* GMI1000. It was found that growth on a small range of metabolites, including dicarboxylates, ethanol, nitrate, ornithine, proline and xanthine, were dependent on only the *rpoN1* gene. Furthermore, the *rpoN1* gene was required for wilt disease on tomato whereas *rpoN2* had no observable role in virulence or metabolism in *R*. *solanacearum* GMI1000. Interestingly, plasmid-based expression of *rpoN2* did not fully rescue the metabolic deficiencies of the Δ*rpoN1* mutants; full recovery was specific to *rpoN1*. In comparison, only *rpoN2* was able to genetically complement a Δ*rpoN E*. *coli* mutant. These results demonstrate that the RpoN1 and RpoN2 proteins are not functionally equivalent or interchangeable in *R*. *solanacearum* GMI1000.

## Introduction

The alternative sigma factor σ^54^ or RpoN regulates an assortment of diverse biological processes and is an essential protein for some bacteria [1]. RpoN proteins are structurally and mechanistically distinct from sigma factors belonging to the σ^70^ family [2]. For example, RpoN proteins have a modular structure consisting of three domains (regions I, II & III). Region I is at the N-terminus and is involved in interactions with transcriptional regulator proteins known as enhancer-binding proteins or EBPs [3–6]. Region II serves as a linker between regions II and III; region II is absent in some RpoN proteins [7]. Region III (C-terminus) is involved in interacting with RNA polymerase [8, 9] and contains the DNA-binding motifs responsible for promoter recognition [9–11]. RpoN recognizes a unique −24/−12 promoter that has a consensus sequence of TGGCACG-N_4_-TTGC [12]. This sequence is remarkably conserved with some nucleotides in the −24 and −12 sites displaying >90% conservation across a number of bacterial species [12].

Our knowledge of RpoN as a regulator of gene expression is largely derived from studies on bacteria harboring a single *rpoN* gene, and thus, a single RpoN protein. Under these circumstances, the RpoN protein has evolved to interact efficiently with its arsenal of partner EBPs to direct transcription from −24/−12 promoters. At the center of these interactions is region I (residues 1–50) of RpoN and the conserved amino acid motif ‘GAFTGA’ localized in a loop structure in the RpoN-interaction domain of EBPs [13]. These two domains make direct contacts with one another, and therefore, play a significant role in allowing RpoN to communicate with EBPs that differ in overall structure and function.

The situation can be more complex when a bacterium possesses multiple RpoN proteins. For example, the photosynthetic bacterium *Rhodobacter sphaeroides* has four genes encoding for RpoN proteins (RpoN1–4), which share 50–60% homology to one another [14]. The RpoN3 and RpoN4 proteins had no observable function in this bacterium [14]. In contrast, RpoN1 was specifically required for transcription of genes associated with nitrogen fixation while RpoN2 was necessary for motility [14]. The specializations of the RpoN1 and RpoN2 proteins were a result of differences in their promoter recognition (at position −11) and EBP interactions [15].

A number of soil-dwelling and plant-associated bacteria do have genes encoding for dual RpoN proteins. For example, the two RpoN proteins in *Bradyrhizobium japonicum* are nearly identical (>80% homology) and were previously found to be interchangeable [16]. The plant pathogen *Ralstonia solanacearum* is widely known for its role as the causative agent of bacterial wilt disease in economically important plant species such as banana, tomato, potato and peanut [17]. The genome of this plant pathogen consists of a 3.7 Mb chromosome and a 2.1 Mb megaplasmid [18]. Interestingly, each of these replicons harbors an *rpoN* gene. The *rpoN1* gene is chromosomal while *rpoN2* is carried on the megaplasmid. Unlike *B*. *japonicum*, the RpoN1 and RpoN2 proteins in *R*. *solanacearum* GMI1000 are not identical. They share ∼60% homology towards one another with the majority of differences being found in regions I and II. Indeed, a recent study showed that the *rpoN* genes in *R*. *solanacearum* GMI1000 are not genetically redundant [19]. Specifically, phenotypic traits such as nitrate utilization, natural competence, twitching motility and virulence were dependent only on the *rpoN1* gene. Inactivation of the *rpoN2* gene had no affect on these phenotypes.

In an effort to expand on these previous findings, and therefore, more fully understand metabolic functions dependent on RpoN in *R*. *solanacearum* GMI1000, we constructed a series of *rpoN*-deletion mutants (Δ*rpoN1*, Δ*rpoN2* and Δ*rpoN1* Δ*rpoN2*) and subsequently tested their growth on an array of compounds. In addition to being essential for nitrate utilization, the *rpoN1* gene was required for the assimilation of dicarboxylates, ethanol, ornithine, proline, propionate and xanthine. We did not observe any metabolic role for the *rpoN2* gene. However, despite the lack of an observable role for metabolic function in *R*. *solanacearum* GMI1000, heterologous expression of the *rpoN2* gene did genetically complement a Δ*rpoN* mutant of *Escherichia coli*. In contrast, heterologous expression of *rpoN1* had no effect on restoring RpoN function in Δ*rpoN E*. *coli*. Plasmid-derived expression of the *rpoN1* gene but not *rpoN2* completely rescued the growth defects of the Δ*rpoN1* and Δ*rpoN1* Δ*rpoN2 R*. *solanacearum* GMI1000 mutants. These findings not only confirm that the *rpoN* genes are not genetically redundant, but importantly, demonstrate that RpoN1 and RpoN2 are not interchangeable (functionally equivalent proteins) in *R*. *solanacearum* GMI1000.

## Materials and Methods

### Bacteria, plasmids and general growth conditions

Bacteria and plasmids used in the study are given in Table 1. *R*. *solanacearum* was maintained in CPG media (1.0 g L^-1^ casamino acids, 10 g L^-1^ peptone, 5 g L^-1^ glucose) [20]. *E*. *coli* was grown in Lennox broth (10 g L^-1^ tryptone, 5 g L^-1^ yeast extract, 5 g L^-1^ NaCl). Antibiotic selection for *R*. *solanacearum* consisted of carbenicillin (Cb) 200 μg mL^-1^, gentamicin (Gm) 15 μg mL^-1^, kanamycin (Km) 50 μg mL^-1^ and tetracycline (Tc) 15 μg mL^-1^. Similar concentrations of antibiotics were used for selection of recombinant *E*. *coli* except for Cb, which was used at 100 μg mL^-1^.

**Table 1:** Bacteria, plasmids and oligonucleotides used in the current study.

Strain, plasmid or oligo-nucleotides		Relevant Characteristics	Source
*Ralstonia solanacearum*			
	GMI1000	wild-type	ATCC
	Δ*rpoN1*	*rpoN1*::Gm^r^	This study
	Δ*rpoN2*	*rpoN2*::Gm^r^	This study
	Δ*rpoN1* Δ*rpoN2*	*rpoN1*::Gm^r^ *rpoN2*::Tc^r^	This study
*Escherichia coli*			
	Top10	F- *mcrA* Δ(*mrr*-*hsdRMS*-*mcrBC*) φ80*lacZ*ΔM15 Δ*lacX74 nupG recA1 araD139* Δ(*ara*-*leu*)7697 *galE15 galK16 rpsL*(Str^R^) *endA1* λ^-^	Invitrogen
	BW25113	F-, *Δ(araD-araB)567*, *ΔlacZ4787*(::rrnB-3), *λ* ^*-*^, *rph-1*, *Δ(rhaD-rhaB)568*, *hsdR514*	[21]
	JM3169-1	F-, *Δ(araD-araB)567*, *ΔlacZ4787*(::rrnB-3), *λ* ^*-*^, *ΔrpoN730*::*kan*, *rph-1*, *Δ(rhaD-rhaB)568*, *hsdR514*	[21]
Plasmids			
	pCR-Blunt	Cloning plasmid; Km^r^	Invitrogen
	pBBR1MCS-2	Broad-host plasmid; Km^r^	[22]
	pDONR221	Cloning plasmid; Km^r^	Invitrogen
	pEX18ApGW	Plasmid for gene deletions in *P*. *aeruginosa*; Cb^r^ Gm^r^	[23]
	pPS856	Plasmid harboring Gm^r^ marker	[24]
	pBR322	Cloning plasmid; Cb^r^ Tc^r^	New England BioLabs
	pBRL533	*rpoN1*::Gm^r^ in pDONR221; Gm^r^ Km^r^	This study
	pBRL534	*rpoN2*::Gm^r^ in pDONR221; Gm^r^ Km^r^	This study
	pBRL535	*rpoN1*::Gm^r^ in pEX18ApGW; Cb^r^ Gm^r^	This study
	pBRL536	*rpoN2*::Gm^r^ in pEX18ApGW; Cb^r^ Gm^r^	This study
	pBRL557	*rpoN2*::Tc^r^ in pDONR221; Gm^r^ Tc^r^	This study
	pBRL560	*rpoN2*::Tc^r^ in pEX18ApGW; Cb^r^ Gm^r^ Tc^r^	This study
	pBRL577	*rpoN1* gene in pCR-Blunt; Km^r^	This study
	pBRL578	*rpoN2* in pCR-Blunt; Km^r^	This study
	pBRL584	*rpoN2* gene in pBBR1MCS-2; Km^r^	This study
	pBRL587	*rpoN1* gene in pBBR1MCS-2; Km^r^	This study
Oligonucleotides			
	BL462.f	5’-tacaaaaaagcaggctatgaaacagtcgctccagctc-3’	
	BL462.r	5’-tcagagcgcttttgaagctaattcggtagtcgctgtcgaaatcgctg-3’	
	BL463.f	5’-aggaacttcaagatccccaattcggagctttttcacgcacggtg-3’	
	BL463.r	5’-tacaagaaagctgggtctataaagacttgcgcagattc-3’	
	BL464.f	5’-tacaaaaaagcaggctgtcaaagccgctctcgaaatg-3’	
	BL464.r	5’-tcagagcgcttttgaagctaattcgcattcgccgatgtcgctgtc-3’	
	BL464.r2	5’-atcgatgataagctgtcaaacatgacattcgccgatgtcgctgtc-3’	
	BL465.f	5’-aggaacttcaagatccccaattcggccaaaatcaagggcaagtgg-3’	
	BL465.f2	5’-cggattcaccactccaagaattggagccaaaatcaagggcaagtgt-3’	
	BL465.r	5’-tacaagaaagctgggtcctcgatcatctccttgagc-3’	
	B568.f	5’-gcaggtaccgatcgactgctgcagttgtg-3’	
	BL568.r	5’-gcatctagactataaagacttgcgcagattcac-3’	
	BL569.f	5’-gcaggtacccgacatgatcatgtagaaacgg-3’	
	BL569.r	5’-gcatctagatcagatctgccgccggag-3’	
	Gm-F	5’-cgaattagcttcaaaagcgctctga-3’	
	Gm-R	5’-cgaattggggatcttgaagttcct-3’	
	Tc-F	5’-tcatgtttgacagcttatcatcgat-3’	
	Tc-R	5’-tccaattcttggagtggtgaatccg-3’	
	GW-*attB1*	5’-ggggacaagtttgtacaaaaaagcaggct-3’	
	GW-*attB2*	5’-ggggaccactttgtacaagaaagctgggt-3’	

### Electroporation of *R*. *solanacearum*

A single colony of *R*. *solanacearum* was inoculated into 10 mL of CPG medium, and the culture was grown for 24 h at 30°C, 200 rpm. The entire 10 mL culture was centrifuged, and the cells were washed two times (1^st^ wash 5.0 mL, 2^nd^ wash 1.0 mL) with 10% (*v/v*) glycerol. After the second wash, cells were suspended in a final volume of 0.1 mL of 10% (*v/v*) glycerol. The cell suspension was given 0.05–1.0 μg of plasmid DNA and the plasmid-cell mixture was transferred to a 2-mm gap electroporation cuvette. Electroporation was performed at 2500 V in an ECM 399 (Harvard Apparatus). Cells were recovered in 1.0 mL of CPG at 30°C, 200 rpm for 1.0 h. Transformants were selected on CPG supplemented with the appropriate antibiotics.

### Standard DNA procedures

DNA was purified using Promega nucleic acid purification kits. Restriction enzymes, ligases and polymerases were products of New England BioLabs. Phusion polymerase (New England BioLabs) was used for all PCR applications. PCR was done using cycling times and parameters as recommended for the Phusion polymerase. Oligonucleotides used for PCR applications were purchased from Integrated DNA Technologies and are listed in Table 1. Cloned DNA was verified by sequencing (Genewiz).

### Cloning of the *rpoN* genes

The *rpoN1* and *rpoN2* genes were PCR amplified with the primers BL568.f/BL568.r and BL569.f/BL569.r, respectively. The desired *rpoN1* and *rpoN2* PCR products (~1.5 kb) were gel-purified and separately cloned into pCR-Blunt (Invitrogen) according to the manufacturer’s instructions. The *rpoN1* and *rpoN2* genes were then individually subcloned into the *Kpn*I/*Xba*I sites of pBBR1MCS-2 [22] to give pBRL587 and pBRL584, respectively.

### Deletion of *rpoN* genes in *R*. *solanacearum* GMI1000

A gene deletion methodology that was originally developed for *Pseudomonas aeruginosa* [23] was adopted and used to inactivate the *rpoN1* and *rpoN2* genes from *R*. *solanacearum* GMI1000. Construction of the *rpoN* deletion plasmids was done according to previously published procedures [23]. The 5’ and 3’ ends (~250 bp) of the *rpoN1* ORF were PCR amplified with the primers BL462.f/BL462.r and BL463.f/BL463.r, respectively. Similarly, primers BL464.f/BL464.r and BL465.f/BL465.r were used to PCR amplify the 5’ and 3’ ends, respectively, of the *rpoN2* ORF. The Gm^r^ marker was PCR amplified from the pPS856 plasmid [24] with the primers Gm-F/Gm-R. The desired PCR products were gel-purified. The purified 5’ and 3’ end fragments of each *rpoN* ORF were fused to Gm^r^ marker *via* PCR with the primers GW-*attB1*/GW-*attB2*. The resulting *rpoN1*::Gm^r^ and *rpoN2*::Gm^r^ cassettes were gel-purified and individually cloned into pDONR221 (Invitrogen) using BP clonase II (Invitrogen). Lastly, LR clonase II (Invitrogen) was used to shuttle the *rpoN1*::Gm^r^ or *rpoN2*::Gm^r^ cassette from pDONR221 into the gene replacement vector pEX18ApGW [23]. The final pBRL535 and pBRL536 plasmids carried the *rpoN1*::Gm^r^ and *rpoN2*::Gm^r^ cassette, respectively, in pEX18ApGW. For the deletion plasmid pBRL560 (*rpoN2*::Tc^r^ pEX18ApGW), primers BL464.f/BL464.r2 and BL465.f2/BL465.r were used to PCR amplify the 5’ and 3’ ends of the *rpoN2* ORF. The Tc^r^ marker was PCR-amplified from pBR322 (NEB) with the primers Tc-F/Tc-R. Fusion PCR and subcloning of the *rpoN2*::Tc^r^ cassette was done using identical procedures as described above.

To generate the single Δ*rpoN1* and Δ*rpoN2* mutants, 0.5–1.0 μg of pBRL535 or pBRL536 was electroporated into (0.1 mL) *R*. *solanacearum* GMI1000. Following a 1.0 h recovery period (30°C, 200 rpm), the entire culture was plated onto CPG supplemented with Gm. The plates were incubated at 30°C for 48 h. Gm^r^ colonies were patched onto CPG supplemented with either Gm or Cb in order to differentiate from double and single crossovers, respectively. After 48 h at 30°C, patched clones displaying Gm^r^ and Cb^s^ were identified as putative Δ*rpoN* mutants. The *rpoN1* and *rpoN2* loci were PCR amplified from the Δ*rpoN1* and Δ*rpoN2* mutants. PCR products were gel-purified, cloned into pCR-Blunt (Invitrogen) and the inserts present in the recombinant plasmids were sequenced to verify the desired Δ*rpoN* mutation.

A similar procedure was used to create the double Δ*rpoN1* Δ*rpoN2* mutant. Briefly, the pBRL560 plasmid was electroporated into Δ*rpoN1 R*. *solanacearum* GMI1000, and colonies were selected for on CPG supplemented with Gm and Tc. Gm^r^ Tc^r^ colonies were patched onto CPG supplemented with Cb, and colonies displaying Cb^s^ were identified as putative Δ*rpoN1* Δ*rpoN2* mutants. The *rpoN* loci were PCR-amplified, cloned and sequenced to verify the mutations in the double Δ*rpoN1* Δ*rpoN2* mutant.

### Growth of Δ*rpoN* mutants of *R*. *solanacearum* GMI1000 on various carbon, nitrogen and sulfur sources

Growth analyses were done in triplicate. Strains were first grown on solid CPG at 30°C for 48 h. Single colonies were inoculated into 1.0 mL of liquid CPG, and the inoculums were grown at 30°C, 200 rpm for 24 h. Next, 10 μL of a CPG-grown seed culture was used to inoculate 1.0 mL of a low-salt minimal media (15 mM K phosphate, 0.7 mM Na citrate, 0.5 mM MgSO_4_, pH 7.0) [20]. For carbon-source testing, minimal media was supplemented with 20 mM NH_4_Cl and carbon sources were used at a final concentration of 20 mM except for benzaldehyde (5 mM), benzoate (10 mM), phenol (5 mM) and pectin [0.2% (*w/v*)], phenylalanine (5 mM), tryptophan (5 mM) and tyrosine (5 mM). For nitrogen-source testing, minimal media was supplemented with 20 mM glucose and nitrogen sources were used at a final concentration of 20 mM except for phenylalanine (5 mM), tryptophan (5 mM), and xanthine (0.5 mM). Lastly, minimal media consisting of 20 mM glucose, 20 mM NH_4_Cl and 0.5 mM MgCl_2_ (in exchange of MgSO_4_) was used to measure growth on sulfur compounds, which were provided at a final concentration of 0.5 mM. Compounds used in the experiments are listed in Table 2.

**Table 2:** Compounds tested as carbon, nitrogen and sulfur sources for wild-type, Δ*rpoN1*, Δ*rpoN2* and Δ*rpoN1* Δ*rpoN2 R*. *solanacearum* GMI1000.

Growth Experiment	Compounds
Carbon Sources	Acetoin, Alanine^a^ ^,^ ^b^, Arginine^a^ ^,^ ^b^, Asparagine^a^ ^,^ ^b^, Aspartate^a^ ^,^ ^b^, Benzoate, Benzaldehyde, Cysteine^b^ ^,^ ^c^, Ethanol, Ethanolamine^a^, Fumarate, Galactose, Glutamate^a^ ^,^ ^b^, Glucose, Gluconate, Glucouronate, Glutamine^a^ ^,^ ^b^, Glycine^a^, Histidine^a^ ^,^ ^b^, Isoleucine^a^ ^,^ ^b^, α-Ketoglutarate (α-KG), Leucine^a^ ^,^ ^b^, Lysine^a^ ^,^ ^b^, Malate, Malonate, Methionine^b^ ^,^ ^c^, Octanoate, Ornithine^a^ ^,^ ^b^, Pectin, Phenol, Phenylalanine^a^ ^,^ ^b^, Proline^a^ ^,^ ^b^, Propionate, Serine^a^ ^,^ ^b^, Sorbitol, Succinate, Sucrose, Taurine^c^, Threonine^a^ ^,^ ^b^, Tryptophan^a^ ^,^ ^b^, Valine^a^ ^,^ ^b^, Xylose
Nitrogen Sources	Acetamide, Ammonium, Carnitine, Nitrate, Nitrite, Urea, Xanthine
Sulfur Sources	HEPES, Methanesulfonate, MOPS, PIPES, Sulfate

^**a**^Compounds were tested as both sole carbon and sole nitrogen sources.

^b^Only L-amino acids were tested.

^c^Compounds were tested as sole carbon, sole nitrogen and sole sulfur sources.

### Genetic complementation experiments

Growth analyses were done in triplicate. Plasmids carrying *rpoN1* (pBRL587), *rpoN2* (pBRL584) or no insert (pBBR1MCS-2) were electroporated into Δ*rpoN1* and Δ*rpoN1* Δ*rpoN2 R*. *solanacearum* GMI1000. Following initial selection, recombinant strains were grown in 1.0 mL of CPG supplemented with Km at 30°C, 200 rpm for 24 h. Minimal media (1.0 mL) was inoculated with 10 μL of CPG-grown seed culture. For complementation experiments relating to carbon utilization, minimal media was supplemented with 20 mM NH_4_Cl and 20 mM α-KG, ethanol, fumarate, malate, propionate or succinate. For complementation experiments relating to nitrogen utilization, minimal media was supplemented with 20 mM glucose and 20 mM alanine, nitrate, ornithine, proline or serine. Minimal media cultures were grown at 30°C, 200 rpm for 24–96 h.

Δ*rpoN E*. *coli* BW25113 was transformed with pBBR1MCS-2, pBRL584 or pBRL587. Recombinant strains were selected on LB supplemented with Km. Individual colonies were inoculated into 1.0 mL of LB supplemented with Km, and the inoculums were grown at 37°C, 200 rpm for 24 h. Minimal media (1.0 mL) was inoculated with 10 μL of LB-grown seed culture. For carbon utilization experiments, minimal media was supplemented with 20 mM NH_4_Cl and acetoacetate (20 mM) or propionate (20 mM). For nitrogen utilization experiments, minimal media was supplemented with 20 mM glucose and arginine (20 mM), asparagine (20 mM), NH_4_Cl (0.5 mM) or xanthine (0.5 mM). Minimal media cultures were grown at 37°C, 200 rpm for 24–48 h.

### Plant assays

Tomato (*Solanum lycopersicum*) cultivar (cv.) Bonny Best and cv. Hawaii 7996 were grown in growth chambers (Conviron) with 12 h light-and-dark cycles at 25°C with 70% relative humidity. *R*. *solanacearum* strains were grown on solid CPG supplemented with 0.005% (*w/v*) of 2,3,5-tripheny tetrazolium chloride (TTC) at 30°C for 48 h. Single colonies that displayed a virulent morphology (mucoid and pinkish-white in color) [20] were grown in liquid CPG at 30°C for 24 h. Cells from each of these cultures were harvested, washed and then diluted in sterile water to a cell density of 1 x 10^6^ CFU mL^-1^. These inoculums were used for the virulence assays as described below.

Virulence of *R*. *solanacearum* strains for tomato cv. Bonny Best and cv. Hawaii 7996 was determined using standard procedures with some modifications [20, 25]. Briefly, stems of healthy plants were wounded by puncturing into pith at the leaf axial using a disposable pipette tip. A drop of bacterial inoculum (1 x 10^6^ CFU mL^-1^) was applied using a pipet tip, and the tip containing 10 μL of inoculum was left at the puncture site. Infected plants were returned to the growth chamber, and the day temperature was increased to 30°C. After the inoculum was taken up by the plant (10–24 h), the pipet tip was discarded.

Disease symptoms were evaluated daily and scored on a 0–5 disease index scale as follows: 0, healthy or no leaf wilting; 1, 10% of the leaf area wilted; 2, 11 to 40% of the leaf area wilted; 3, 41 to 60% of the leaf area wilted; 4, 61 to 85% of the leaf area wilted, 5, 85% of the leaf area wilted or dead leaf. Disease wilting index (dwi) was calculated using the following formula [20]: dwi (%) = [(sum of rating 0–5)] x 0.2 x (number of leaves evaluated) x 100. Plant assays were performed at least in duplicates using 8–9 plants per treatment.

The plants were sampled at 4 d post inoculation (dpi) for cv. Bonny Best and 6–7 dpi for cv. Hawaii7996. One-centimeter stem pieces just above the infection site were cut using a sterile razor blade. The stem pieces were weighed, sterilized with 70% (*v/v*) ethanol and then rinsed twice with sterile water. The stem pieces were ground using pestle in 800 μL of sterile water and serial dilutions were plated on CPG supplemented with 0.005% (*w/v*) TTC. Plates were incubated at 30°C for 18–24 h, and the number of bacterial colonies present were recorded. All experiments were performed at least in duplicates for each bacterial stain for both tomato cultivars. Data was analyzed using student t-tests, and values reported represent a mean of the colonies (± SD) per gram of fresh tissue.

## Results and Discussion

### Assimilation of a limited but diverse group of compounds is dependent only on the *rpoN1* gene in *R*. *solanacearum* GMI1000

RpoN is well known for being involved in the assimilation of a wide variety of compounds among bacteria. Therefore, the Δ*rpoN1*, Δ*rpoN2* and Δ*rpoN1* Δ*rpoN2* mutants of *R*. *solanacearum* GMI1000 were challenged with an array of carbon, nitrogen and sulfur sources (Table 2). Preliminary experiments showed that all three *rpoN* mutants grew to cell densities (OD_600nm_ values) equivalent to that of wild-type in minimal media supplemented with 20 mM glucose and 20 mM NH_4_Cl (S1 Table). Based on this result, glucose was chosen as the carbon source for nitrogen utilization experiments while NH_4_Cl served as the nitrogen source for carbon utilization experiments.

**Fig 1:**
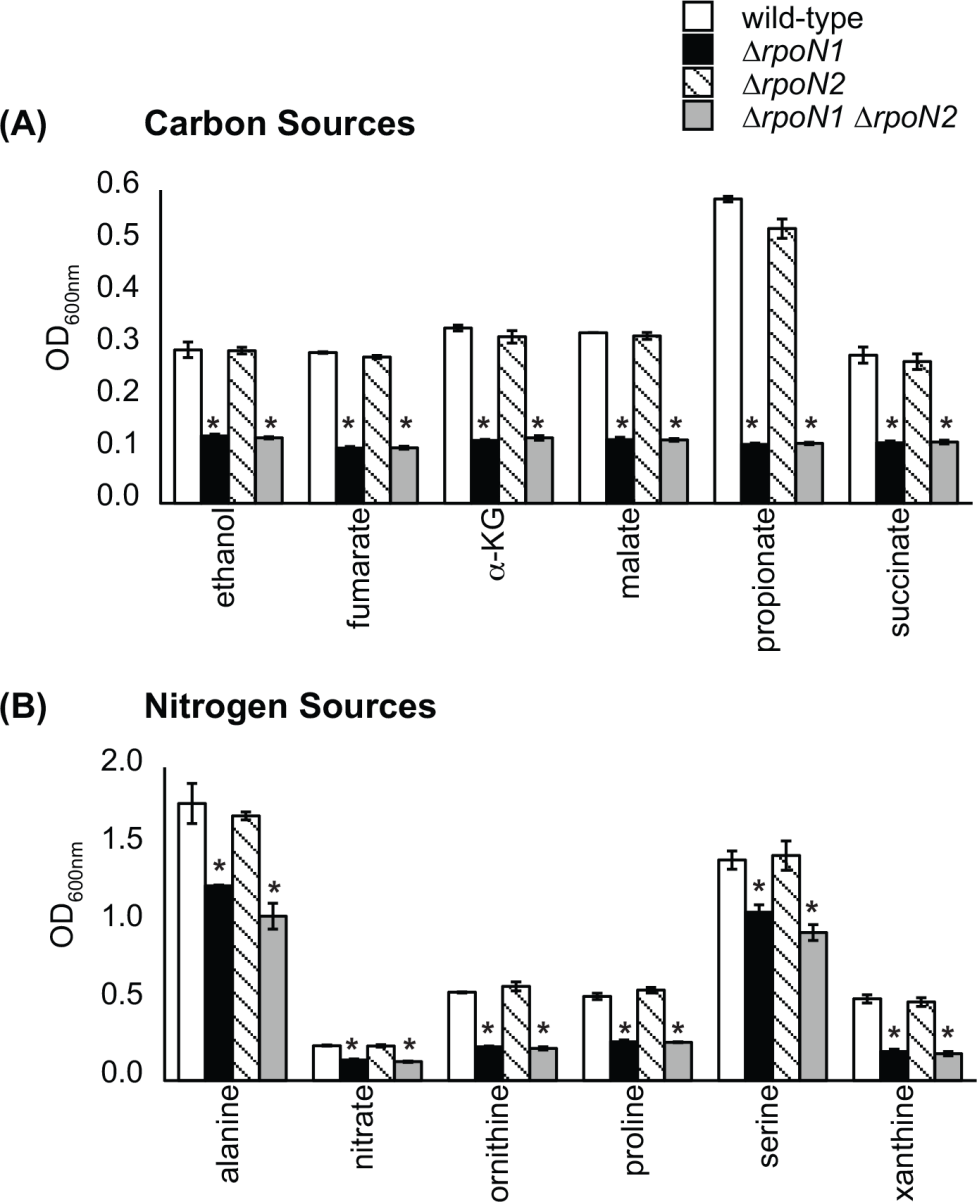
The *rpoN1* gene was required for growth on a small number of compounds. (A) The Δ*rpoN1* and Δ*rpoN1* Δ*rpoN2* mutants failed to grow on C_4_-dicarboxylates, ethanol, α-KG and propionate when provided as sole carbon sources. (B) The Δ*rpoN1* and Δ*rpoN1* Δ*rpoN2* mutants grew poorly when alanine, nitrate, ornithine, proline, serine and xanthine were provided as sole nitrogen sources. Out of the total >50 compounds tested, the utilization of only a dozen of them were found to require the *rpoN1* gene in *R*. *solanacearum* GMI1000. [Data points represent mean values (n = 3) ± SD. Analysis of variance was done using Dunnett’s post hoc test (α-value of 0.05) to identify significant changes (*P* < 0.0001), which are marked with an asterisk].

Surprisingly, only the Δ*rpoN1* and Δ*rpoN1* Δ*rpoN2* mutants were observed to have either zero or reduced growth on a limited number of compounds (Fig 1). The Δ*rpoN1* and Δ*rpoN1* Δ*rpoN2* mutants could not grow on ethanol, propionate, fumarate, malate, succinate and α-KG as carbon sources. For nitrogen sources, nitrate, ornithine, proline and xanthine did not support the growth of the Δ*rpoN1* and Δ*rpoN1* Δ*rpoN2* mutants. The Δ*rpoN1* and Δ*rpoN1* Δ*rpoN2* mutants had decreased growth on alanine and serine when provided as nitrogen sources. The Δ*rpoN2* mutant exhibited wild-type growth on all compounds. Furthermore, all *rpoN* mutants showed wild-type growth on aliphatic sulfur sources even though RpoN is known to regulate such assimilation in other bacteria (S1 Table) [26].

The metabolic deficiencies of the Δ*rpoN1* and Δ*rpoN1* Δ*rpoN2* mutants are in agreement with the putative EBPs and RpoN-controlled genes of *R*. *solanacearum* GMI1000 (Table 3). For example, the *rpoN1* gene was necessary for the growth of *R*. *solanacearum* GMI1000 on C_4_-dicarboxylates and α-KG. In a number of bacteria, dicarboxylate utilization is dependent on RpoN and an EBP known as DctD, which together activate transcription of genes encoding for dicarboxylate transport proteins [27–29]. In *R*. *solanacearum* GMI1000, the *Rsp0009* (*dctD1*) and *Rsp0332* (*dctD2*) genes encode for homologs of DctD, and RpoN promoters are found upstream of genes encoding for C_4_-dicarboxylates and α-KG transport proteins. It is expected that *R*. *solanacearum* GMI1000 also uses an RpoN-DctD mechanism to regulate uptake of dicarboxylates.

**Table 3:** EBPs and their potential target genes of *R*. *solanacearum* GMI1000.

EBP		Putative function	Potential regulated genes having RpoN promoters	
Symbol	Name		Symbol	Function
*Rsc0222* ^*a*^ ^,^ ^b^	*rtcR*	regulator of *rtcB*	*Rsc0224*	RNA ligase (RtcB)
*Rsc0332* ^b^	*dctD2*	dicarboxylate transport	*Rsc0330*	C_4_-dicarboxylate transporter (DctA)
*Rsc1186* ^b^ ^,^ ^c^	–	–	–	–
*Rsc1261* ^b^ ^,^ ^d^	*ntrC*	nitrogen assimilation	*RSc0381*	nitrate transporter (NasF)
			*Rsc1258*	glutamine synthetase (GlnA1)
			*RSc2118*	xanthine permease
			*Rsc3410*	amino acid-binding periplasmic protein
			*Rsp0886*	glutamine synthetase (GlnA2)
			*Rsp0942*	nitrogen assimilation transcriptional regulator (Nac)
			*Rsp1671*	RpoN2
			*Rsp1223*	nitrate transporter
*Rsc2807* ^b^ ^,^ ^e^	*pehR*	type IV pilibiosynthesis	*Rsc0558*	fimbrial pilin (PilA)
			*Rsc2827*	prepilin peptidase/methyltransferase protein (PilD)
*Rsc3129* ^f^	*acoR*	ethanol catabolism	*Rsc3128*	acetaldehyde dehydrogenase (ExaC)
*Rsp0009* ^b^	*dctD1*	dicarboxylate transport	*Rsp0007*	α-ketoglutarate permease (KgtP2)
*Rsp0123* ^b^	*prpR*	propionate catabolism	*Rsp0122*	2-methylisocitrate lyase (PrpB)
*Rsp0228* ^g^	–	–	*Rsp0229*	benzaldehyde dehydrogenase oxidoreductase
*Rsp0959* ^b^	–	–	*Rsp0958*	iron-sulfur cluster repair protein
*Rsp1079* ^b^	–	–	*Rsp1076*	glucose-fructose oxidoreductase
*Rsp1667* ^f^	–	–	*Rps1668*	EBP
			*Rsp1671*	RpoN2
*Rsp1668* ^f^	–	–	*Rsp1668*	EBP
			*Rsp1671*	RpoN2

Because the RpoN-interaction domain is conserved in EBPs, the RpoN-interaction domain of *E*. *coli* NtrC was used in a BlastP search against the protein database of *R*. *solanacearum* GMI1000. This searched returned a total of thirteen putative EBPs, which is in agreement with a previously published assessment [30]. Nine EBPs possess the signature GAFTGA motif while the remaining four have GSFTGA or GAYTGA. A partial listing of potential gene targets regulated by RpoN and EBPs are given. Full listing of genes harboring putative RpoN promoters can be accessed and searched in the Sigma 54 Database (www.sigma54.ca) [31].

^a.^ “*Rsc*” are chromosomal genes while “*Rsp*” indicates genes carried on megaplasmid.

^b.^ EBP has GAFTGA motif in RpoN-interaction domain.

^c.^ There are no genes near this EBP that possess RpoN promoters.

^d.^ NtrC might regulate numerous genes. Only a select few are shown.

^e.^ PehR shares homology with the pili-biosynthesis regulator EBP PilR. *pehR* mutants have been observed to be defective in type IV pili and twitching motility [19, 32].

^f.^ EBP has GSFTGA motif in RpoN-interaction domain.

^g.^ EBP has GAYTGA motif in RpoN-interaction domain.

RpoN and the EBP PrpR regulate the degradation of propionate in some bacteria [33, 34]. Indeed, the Δ*rpoN1* and Δ*rpoN1* Δ*rpoN2* mutants of *R*. *solanacearum* GMI1000 did not grow on propionate as a carbon source. Rsp0123 is a predicted homolog of PrpR, and there is an RpoN promoter upstream of an operon encoding for enzymes of a propionate catabolic pathway in *R*. *solanacearum* GMI1000. Propionate catabolism in *R*. *solanacearum* GMI1000 is likely regulated by RpoN1 and PrpR. Rsc3129 has homology to the EBP AcoR [35, 36] and is predicted to regulate transcription of an acetaldehyde dehydrogenase (ExaC), a key enzyme in the breakdown of acetoin, ethanol and ethanolamine [37, 38]. The *rpoN1* gene was required for growth on ethanol, but we observed no growth for the wild-type strain or *rpoN* mutants on acetoin or ethanolamine.

Pectin, sucrose, galactose and hexoses in general are common substrates for *R*. *solanacearum* in the environment. Deletion of the *rpoN* genes had no impact on the consumption of these compounds. The utilization of nitrate, however, was completely dependent on the *rpoN1* gene. This finding is in agreement with a previous study [19]. Nitrate assimilation was observed to be a crucial component for *R*. *solanacearum* in the host plant environment [39]. The NtrC-response was involved in nitrate utilization [39]. Nitrate might serve as a valuable nitrogen source for *R*. *solanacearum* during host plant interactions.

The *rpoN* genes were not required for the use of amino acids as carbon sources. However, ornithine and proline were the only amino acids that could not be used as nitrogen sources by the Δ*rpoN1* mutants. The Δ*rpoN1* mutants grew poorly on serine and alanine as nitrogen sources but nonetheless still produced wild-type cell densities after 72 h of incubation. The strict *rpoN1*-dependency for ornithine could be attributed to the Rsc3410 locus, which encodes for a protein sharing some homology to the glutamine transporter GlnH [40]. Ornithine transport might be mediated through Rsc3410. There are no putative RpoN-controlled genes encoding for catabolic enzymes of proline degradation in *R*. *solanacearum* GMI1000. Proline degradation is regulated by Nac (nitrogen assimilation regulator) in some bacteria [41, 42], and the *nac* gene in *R*. *solanacearum* GMI1000 is preceded by an RpoN promoter. This might account for the RpoN1-dependency of proline utilization.

### RpoN1 and RpoN2 are not interchangeable

**Fig 2:**
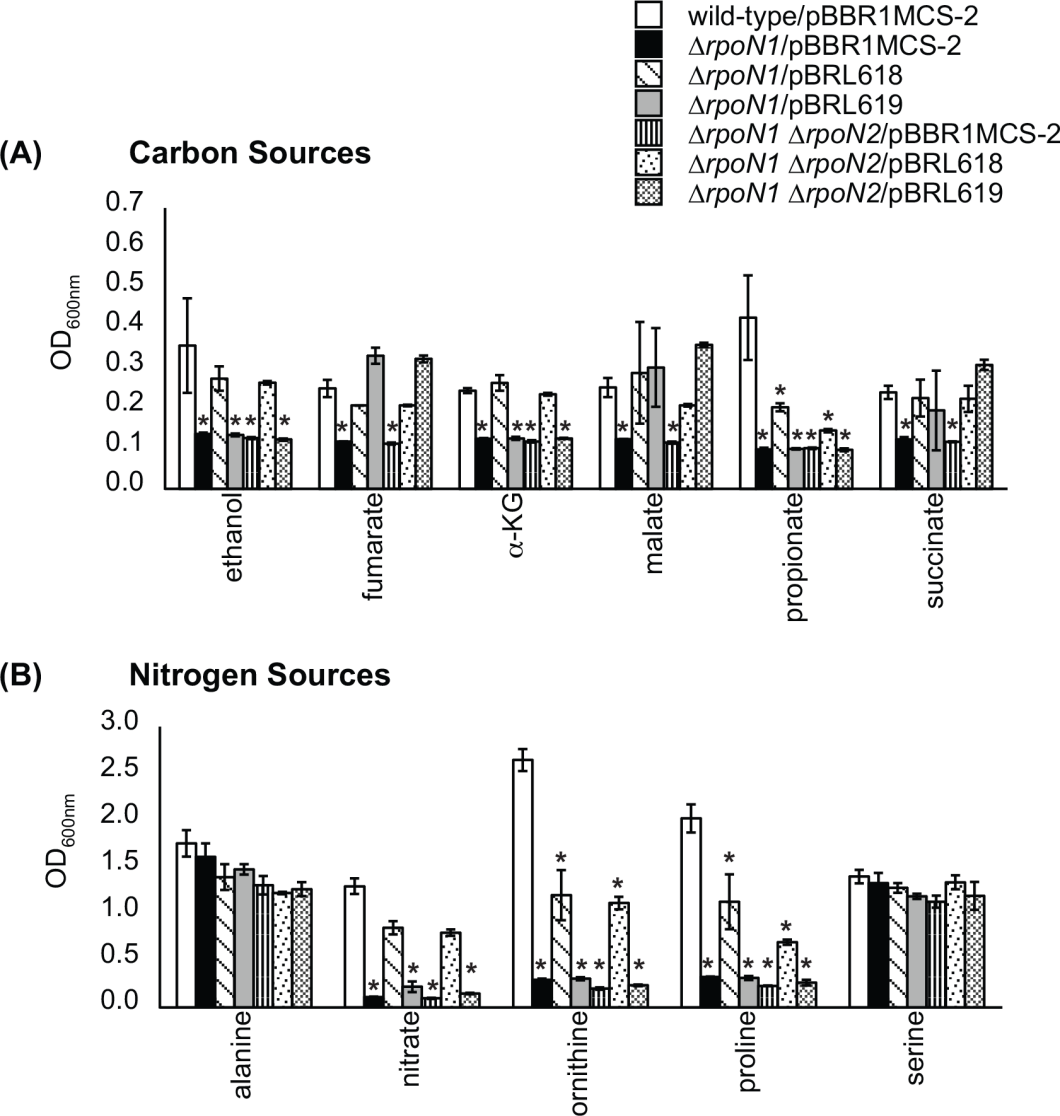
Plasmid-derived expression of *rpoN2* restored the growth of the Δ*rpoN1* mutants on C_4_-dicarboxylates. (A) Plasmid-derived expression of *rpoN1* (pBRL618) or *rpoN2* (pBRL619) enabled the Δ*rpoN1* and Δ*rpoN1* Δ*rpoN2* mutants to grow on C_4_-dicarboxylates as carbon sources. In contrast, expression of *rpoN1* but not *rpoN2* rescued the growth of the Δ*rpoN1* and Δ*rpoN1* Δ*rpoN2* mutants on ethanol, α-KG and propionate. (B) Plasmid-derived expression of *rpoN1* allowed for the utilization of nitrate, ornithine and proline as nitrogen sources in the Δ*rpoN1* and Δ*rpoN1* Δ*rpoN2* mutants. The inability of *rpoN2* expression to completely genetically complement the Δ*rpoN1* and Δ*rpoN1* Δ*rpoN2* mutants is suggestive that the RpoN1 and RpoN2 proteins are not functionally equivalent. Note that we did not observe full recovery for some substrates. The *rpoN* genes were expressed from the *lac* promoter of pBBR1MCS-2. The weakness of the *lac* promoter might keep RpoN protein levels below what is needed for full complementation. [Data points represent mean values (n = 3) ± SD. Analysis of variance was done using Dunnett’s post hoc test (α-value of 0.05) to identify significant changes (*P* < 0.0001), which are marked with an asterisk].

The *rpoN1* and *rpoN2* genes were individually cloned under the *lac*-promoter on the broad-host range plasmid pBBR1MCS-2 [22]. The plasmid-encoded *rpoN1* and *rpoN2* genes were electroporated into the Δ*rpoN1* and Δ*rpoN1* Δ*rpoN2* mutants, and the recombinant strains were challenged with compounds whose assimilation was previously found to be *rpoN1*-dependent. As shown in Fig 2, plasmid-derived expression of either *rpoN1* or *rpoN2* recovered the growth of the Δ*rpoN1* and Δ*rpoN1* Δ*rpoN2* mutants on C_4_-dicarboxylates. Additionally, expression of *rpoN1* rescued the growth of the Δ*rpoN1* and Δ*rpoN1* Δ*rpoN2* mutants on ethanol, α-KG, nitrate, ornithine, proline and propionate. In sharp contrast, expression of *rpoN2* did not allow for the growth of the Δ*rpoN1* and Δ*rpoN1* Δ*rpoN2* mutants on these compounds. Complementation *via* RpoN2 was specific for C_4_-dicarboxylates, indicating that RpoN1 and RpoN2 are not equivalent proteins. Although this observation was unexpected at first, subsequent studies with the *rpoN1*/*rpoN2* genes in Δ*rpoN E*. *coli* confirmed that the RpoN1 and RpoN2 proteins are not functionally identical.

### RpoN1 and RpoN2 have different functionality in Δ*rpoN E*. *coli*

The RpoN1 and RpoN2 proteins share 63% and 55% homology to *E*. *coli* RpoN, respectively. Therefore, we decided to determine if the *rpoN1* and *rpoN2* genes were capable of genetically complementing a Δ*rpoN E*. *coli* strain [21]. The plasmid-encoded *rpoN1* and *rpoN2* genes were introduced into Δ*rpoN E*. *coli* strain, and the recombinant Δ*rpoN E*. *coli* strains were assayed under growth conditions known to be RpoN-dependent, including nitrogen assimilation (NtrC-regulated response) [43], propionate catabolism (PrpR-regulated response) [33] and acetoacetate metabolism (AtoC-regulated response) [44].

**Fig 3:**
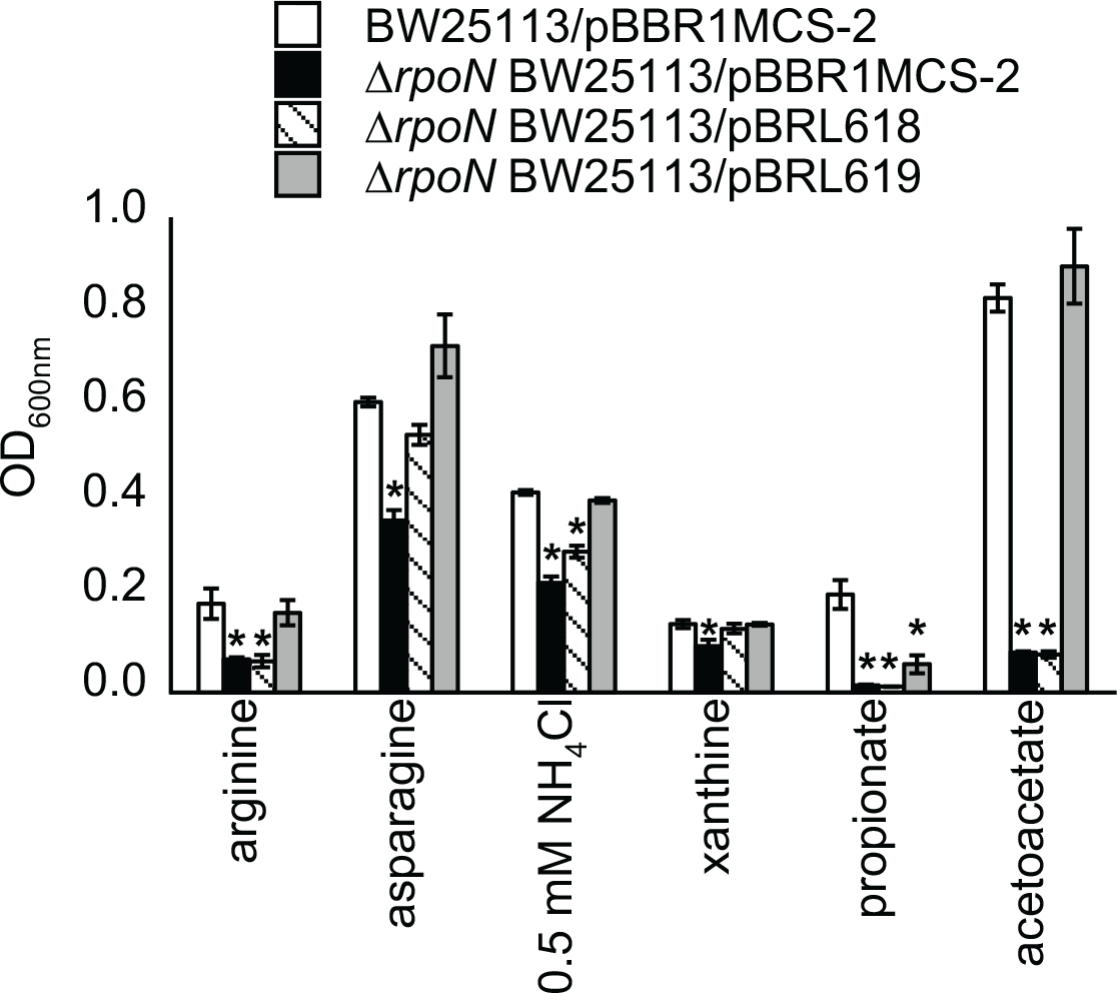
RpoN1 and RpoN2 displayed different properties in Δ*rpoN E*. *coli*. Plasmids carrying *rpoN1* (pBRL618), *rpoN2* (pBRL619) and no insert (pBBR1MCS-2) were introduced into Δ*rpoN E*. *coli* BW25113. As shown, expression of *rpoN2* (and not *rpoN1*) restored the growth of Δ*rpoN E*. *coli* under conditions known to be RpoN dependent, including nitrogen limitation (0.5 mM NH_4_Cl), assimilation of nitrogenous compounds (arginine, asparagine and xanthine), acetoacetate utilization and propionate catabolism. RpoN2 and *E*. *coli* RpoN share weak homology in region II, which might account for RpoN2 being able to complement the Δ*rpoN* mutant. [Data points represent mean values (n = 3) ± SD. Analysis was done using Dunnett’s post hoc test (α-value of 0.05) to identify significant changes (*P* < 0.0001), which are marked with an asterisk].

Heterologous expression of either the *rpoN1* or *rpoN2* gene did recover the growth of the Δ*rpoN E*. *coli* on nitrogen sources such as asparagine and xanthine (Fig 3). However, only the expression of *rpoN2* completely rescued the growth of Δ*rpoN E*. *coli* under nitrogen limitation (NH_4_ < 0.5mM) and when arginine was the sole nitrogen source. Additionally, *rpoN2* (but not *rpoN1*) restored the growth of Δ*rpoN E*. *coli* on propionate and acetoacetate to ∼25% and 100% of wild-type levels, respectively. These results indicate that the RpoN1 and RpoN2 proteins are not functionally equivalent.

**Fig 4:**
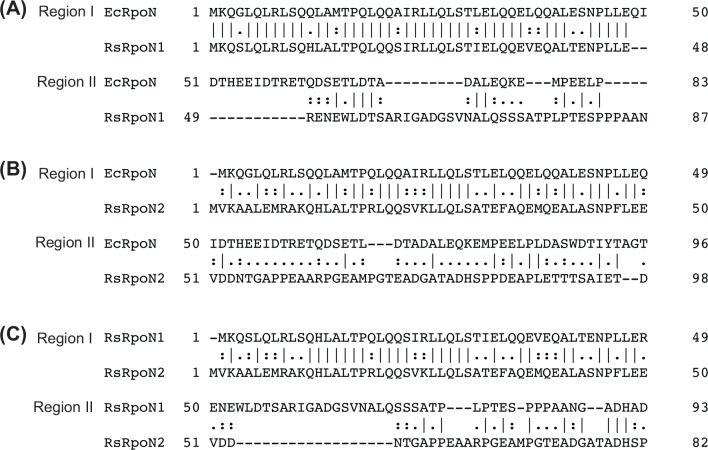
Alignment of regions I (residues 1–50) and II (residues 51–100) of *E*. *coli* RpoN with RpoN1 and RpoN2 of *R*. *solanacearum* GMI1000. (A) *E*. *coli* RpoN (EcRpoN) and RpoN1 of *R*. *solanacearum* GMI1000 (RsRpoN1) (B) EcRpoN and *R*. *solanacearum* GMI1000 RpoN2 (RsRpoN2). (C) RsRpoN1 and RsRpoN2. *E*. *coli* RpoN and RpoN2 have homology in region II, which might enable RpoN2 to interact with EBPs of *E*. *coli*. Alignments were generated using EMBOSS.

Alignment of RpoN1, RpoN2 and *E*. *coli* RpoN revealed that all three proteins have ∼80% homology in region I (residues 1–50) and 60% homology in region III (C-terminal 350 residues). For RpoN2 and *E*. *coli* RpoN, they also share weak homology in region II (residues 51–100) (Fig 4). This homology does not exist between RpoN1 and *E*. *coli* RpoN, and thus, would explain why RpoN2 was more sufficient in complementing Δ*rpoN E*. *coli* than that of RpoN1 (Fig 3).

Region II of RpoN has been implicated in EBP interactions [7], and NtrC of *R*. *solanacearum* GMI1000 has 70% homology to *E*. *coli* NtrC. Based on our results, it would appear that RpoN2 could function with NtrC of *E*. *coli* to facilitate nitrogen assimilation. RpoN1 can also interact with *E*. *coli* NtrC but to a much lesser extent than that of RpoN2 [as judged by RpoN1 being able to only *partially* compensate for nitrogen assimilation in Δ*rpoN E*. *coli*]. *R*. *solanacearum* NtrC exhibits 60% homology to *E*. *coli* AtoC. Expression of *rpoN2* restored the growth of Δ*rpoN E*. *coli* on acetoacetate, suggesting that RpoN2 can partner with AtoC to regulate transcription from AtoC-controlled genes. *R*. *solanacearum* PrpR and *E*. *coli* PrpR have ∼50% homology. The differences in sequence between the PrpR EBPs are reflected in the inability of RpoN2 to fully restore the growth of Δ*rpoN E*. *coli* on propionate.

### The *rpoN1* gene is required for virulence of *R*. *solanacearum* GMI1000 on tomato

**Fig 5:**
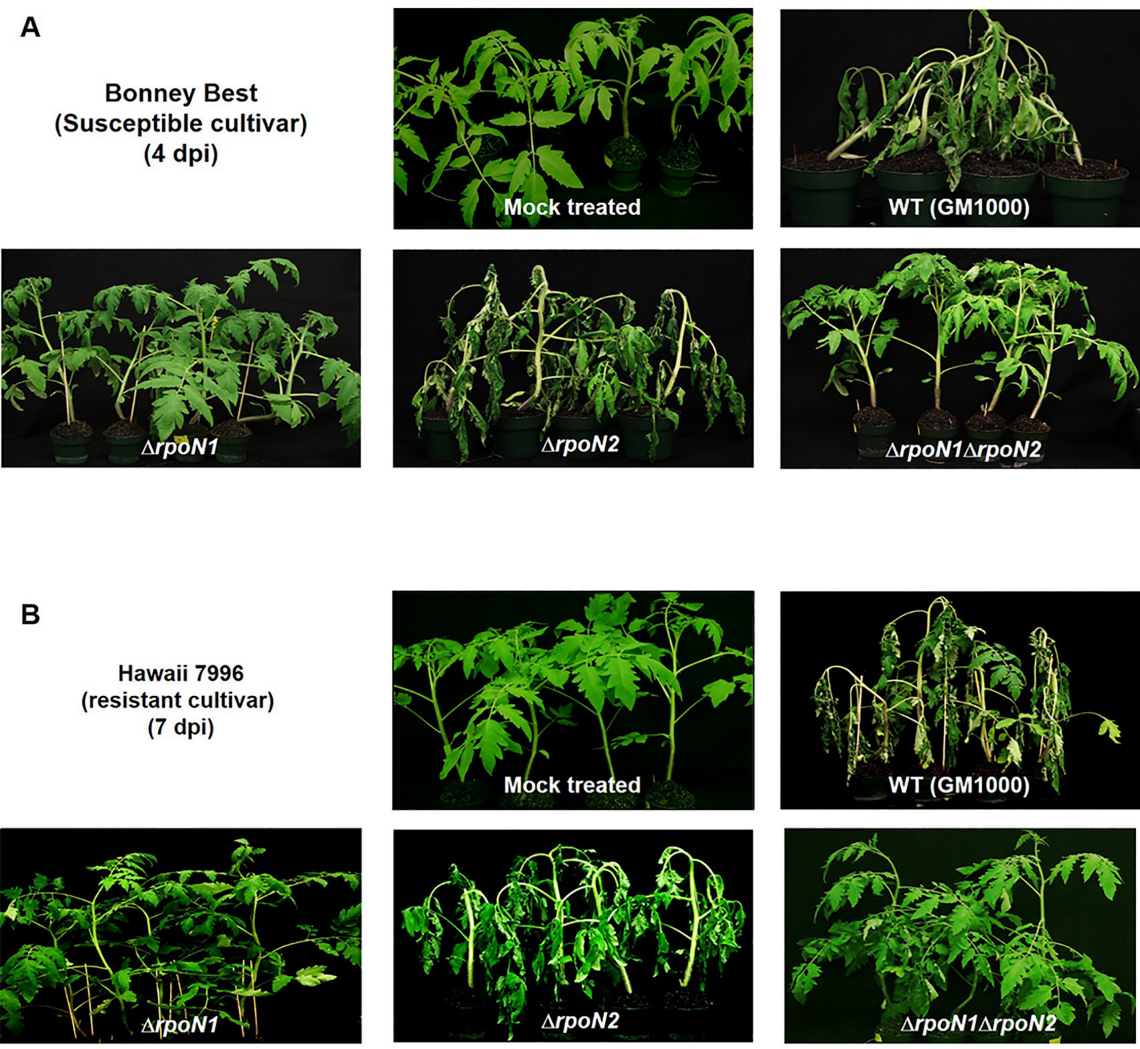
The *rpoN1* gene was required for wilt disease on tomato. (A) cv. Bonny Best at 4 dpi. (B) cv. Hawaii 7996 at 7 dpi. Tomato plants infected with the Δ*rpoN1* and Δ*rpoN1* Δ*rpoN2* mutants did not show signs of wilting disease compared to the Δ*rpoN2* mutant and wild-type *R*. *solanacearum* GMI1000.

**Fig 6:**
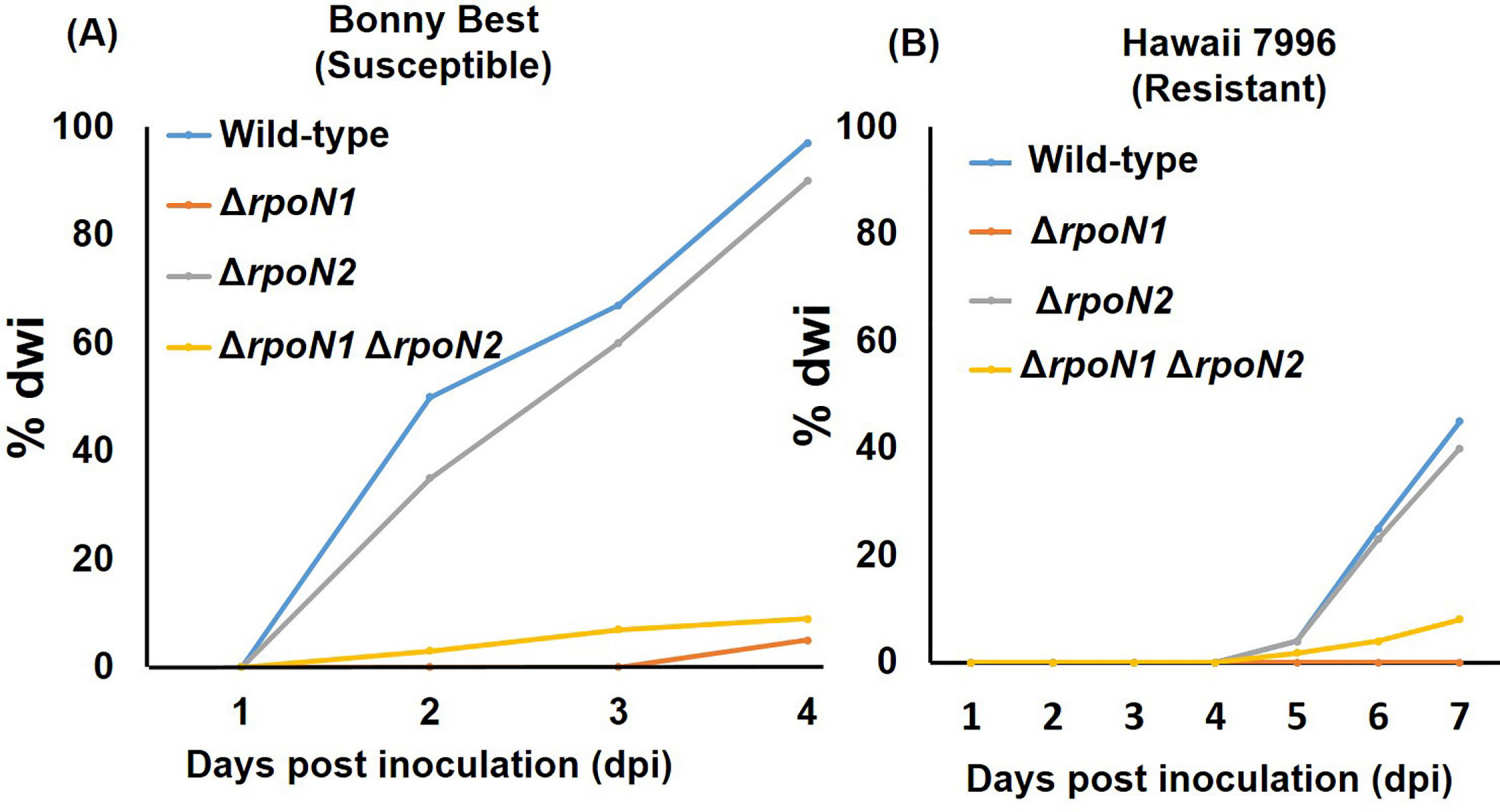
Quantification of wilt disease caused by wild-type, Δ*rpoN1*, Δ*rpoN2* and Δ*rpoN1* Δ*rpoN2 R*. *solanacearum* GMI1000 on tomato. (A) cv. Bonny Best. (B) cv. Hawaii 7996. Wilt symptoms were rated daily on a disease index scale, and the values were used to calculate a disease-wilting index (dwi). The dwi for both the Δ*rpoN1* and Δ*rpoN1* Δ*rpoN2* mutant was significantly reduced compared to the Δ*rpoN2* mutant and wild-type *R*. *solanacearum* GMI1000. Note that nine plants were used for each treatment.

Dicarboxylates and nitrate were previously implicated as key nutrients for *R*. *solanacearum* in the host plant environment [25, 39]. Because the *rpoN1* gene was needed for the growth of *R*. *solanacearum* GMI1000 on these compounds, it was suspected that *rpoN1* might also be a necessary factor in the pathogenesis of this pathogen. Indeed, this suspicion was recently confirmed in a study, which found that the *rpoN1* gene was necessary for virulence of *R*. *solanacearum* GMI1000 on tomato plants (*Lycopersicum esculentum* cv. Marmande)[19]. Therefore, we decided to determine if the Δ*rpoN1* mutants generated in our study were also avirulent. To this end, tomato plants consisting of cv. Bonny Best (susceptible) and cv. Hawaii 7996 (resistant) were inoculated with a bacterial suspension comprising of wild-type, Δ*rpoN1*, Δ*rpoN2* or Δ*rpoN1* Δ*rpoN2 R*. *solanacearum* GMI1000. The inoculated plants were then examined on a daily basis for signs of wilt disease, which was quantified and used to calculate a disease-wilting index (dwi). Wilting was first observed on the older leaves of cv. Bonny Best plants inoculated with wild-type and Δ*rpoN2 R*. *solanacearum* GMI1000. The overall pattern of disease was similar for both tomato cultivars but the extent of wilting was severe in cv. Bonny Best compared to cv. Hawaii 7996 (Fig 5). Entire cv. Bonny Best plants wilted earlier (4 dpi) than cv. Hawaii 7996 (7 dpi). However, Δ*rpoN1-* and Δ*rpoN1* Δ*rpoN2-*inoculated cv. Bonny Best and cv. Hawaii 7996 plants did not develop any significant wilting symptoms after 4 and 7 days, respectively (Fig 5). Mock plants treated with sterile water had no wilting symptoms (Fig 5). At 2 dpi, wild-type and Δ*rpoN2*-inoculated cv. Bonny Best plants had 35–50% dwi, which increased to 60–67% at 3 dpi. In contrast, progression of disease was reduced in Δ*rpoN1*- and Δ*rpoN1* Δ*rpoN2*-inoculated cv. Bonny Best plants with dwi reaching only up to 0–7% at 3 dpi (Fig 6A). The wild-type and Δ*rpoN2* inoculated cv. Hawaii 7996 population had 23–45% dwi at 6 dpi, which increased up to 40–45% at 7 dpi. Δ*rpoN1*- and Δ*rpoN1* Δ*rpoN2*-inoculated cv. Hawaii-7996 population did not show any visible disease symptoms. At 6 dpi, the dwi for these plants was 0–4%, which did not significantly change at 7 dpi (0–8%) (Fig 6B).

**Fig 7:**
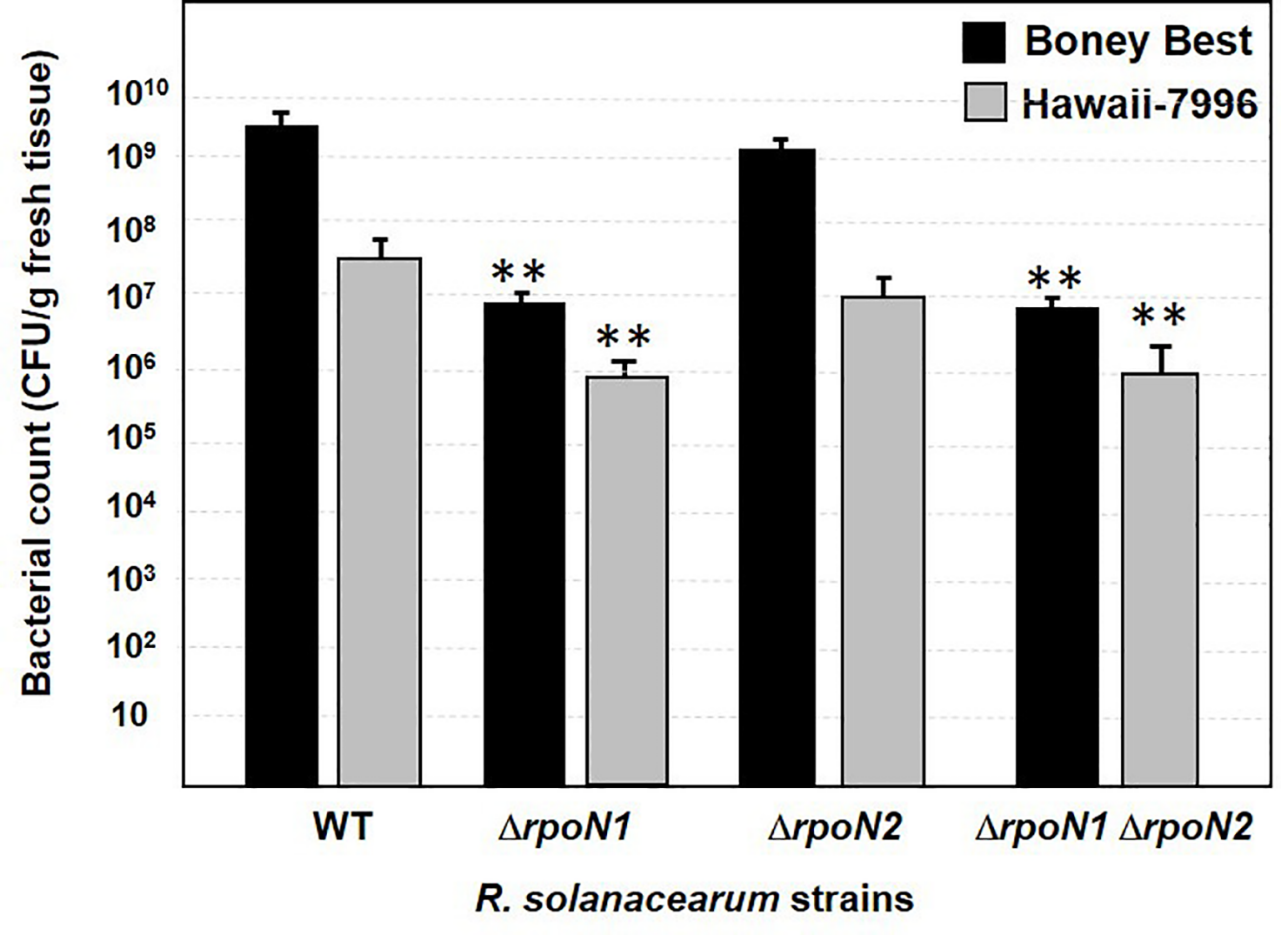
*In planta* growth of wild type, Δ*rpoN1*, Δ*rpoN2* and Δ*rpoN1* Δ*rpoN2 R*. *solanacearum* GMI1000 in tomato. Bacterial growth (CFUs per gram of fresh tissue) was determined at 4 dpi in cv. Bonny Best and 7 dpi in cv. Hawaii 7996. For both tomato cultivars, the Δ*rpoN1* and Δ*rpoN1* Δ*rpoN2* mutants yielded lower CFUs (reduced growth) compared to the Δ*rpoN2* mutant and wild-type *R*. *solanacearum* GMI1000. [Data points represent mean values (n = 8) ± SD. Analysis was done using Student t-test to identify significant changes (*P* < 0.005), which are marked with double asterisk].

We next evaluated the *in planta* growth of the *rpoN* mutants. Plants were inoculated with each strain at a titer of 1 x 10^6^ CFU mL^-1^ and bacterial growth (CFUs per gram of fresh tissue) was determined at 4 dpi for cv. Bonny Best and 7 dpi for cv. Hawaii 7996. The bacterial growth could not be determined at 7 dpi for the cv. Bonny Best plants, because all plants were dead by this particular dpi. At 4 dpi in cv. Bonny Best, wild-type cells and the Δ*rpoN2* mutant produced ~1000-fold more CFUs than that of the Δ*rpoN1* and Δ*rpoN1* Δ*rpoN2* mutants. Similarly, at 7 dpi in cv. Hawaii 7996, wild-type cells and the Δ*rpoN2* mutant produced ~100-fold more CFUs than that of the Δ*rpoN1* and Δ*rpoN1* Δ*rpoN2* mutants (Fig 7). These results are consistent with the patterns of wilting disease caused by wild-type and Δ*rpoN2* strains on both tomato cultivars. Collectively, these findings demonstrate that *rpoN1* but not *rpoN2* is critical for virulence of *R*. *solanacearum* on tomato and they are not functionally redundant for virulence.

## Conclusions

Although two *rpoN* genes are present in *R*. *solanacearum* GMI1000, our results, in combination with previous findings clearly indicate that the *rpoN1* and *rpoN2* genes are not genetically redundant. Functions commonly associated with RpoN regulation were dependent only on the *rpoN1* gene. Additionally, this study demonstrates that the RpoN1 and RpoN2 proteins are not equivalent or interchangeable. Why *R*. *solanacearum* GMI1000 possesses the genetic machinery to encode dual RpoN proteins that are not interchangeable is an intriguing question. It is possible that the EBPs of *R*. *solanacearum* GMI1000 have evolved to preferentially interact with RpoN1 to activate transcription from RpoN promoters while RpoN2 may have evolved to play more of an antagonistic role in *R*. *solanacearum* GMI1000, *i*.*e*., repressing transcription from genes possessing RpoN promoters. Future studies aimed at identifying the RpoN-EBP partnerships in *R*. *solanacearum* GMI1000 are expected to clarify the regulatory roles of the RpoN1 and RpoN2 proteins.

## Supporting Information

S1 Table Growth data of wild-type, Δ*rpoN1*, Δ*rpoN2*, Δ*rpoN1* Δ*rpoN2 R*. *solanacearum* GMI1000 on various carbon, nitrogen and sulfur sources. Each strain (n = 3) was grown in minimal media in which the indicated compound served as either the sole carbon, nitrogen or sulfur source. The mean absorbance value at 600 nm (± standard deviation) is reported for each strain.
(DOCX)
