# Supplementary material for: Defining the Metabolic Functions and Roles in Virulence of the rpoN1 and rpoN2 Genes in Ralstonia solanacearum GMI1000
Source: PLoS One. 2015 Dec 11;10(12):e0144852. doi: 10.1371/journal.pone.0144852 (PMC4676750; doi:10.1371/journal.pone.0144852)
Supplement: S1 Table — Each strain (n = 3) was grown in minimal media in which the indicated compound served as either the sole carbon, nitrogen or sulfur source. The mean absorbance value at 600 nm (± standard deviation) is reported for each strain. (DOCX) [file pone.0144852.s001.docx]

**S1 Table. Growth data of wild-type, ∆*rpoN1*, ∆*rpoN2*, ∆*rpoN1* ∆*rpoN2* *R. solanacearum* GMI1000 on various carbon, nitrogen and sulfur sources.** Each strain (n = 3) was grown in minimal media in which the indicated compound served as either the sole carbon, nitrogen or sulfur source. The mean absorbance value at 600 nm (± standard deviation) is reported for each strain.

|  | **wild-type** | **∆*rpoN1*** | **∆*rpoN2*** | **∆*rpoN1* ∆*rpoN2*** |
| --- | --- | --- | --- | --- |
| **Carbon** |  |  |  |  |
| acetoin | 0.102 ± 0.002 | 0.109 ± 0.003 | 0.100 ± 0.001 | 0.108 ± 0.002 |
| alanine | 0.508 ± 0.020 | 0.430 ± 0.021 | 0.505 ± 0.013 | 0.428 ± 0.016 |
| arginine | 0.113 ± 0.003 | 0.115 ± 0.001 | 0.116 ± 0.007 | 0.129 ± 0.002 |
| asparagine | 0.300 ± 0.017 | 0.264 ± 0.020 | 0.283 ± 0.009 | 0.269 ± 0.003 |
| aspartate | 0.326 ± 0.011 | 0.325 ± 0.005 | 0.328 ± 0.008 | 0.386 ± 0.011 |
| benzoate | 0.039 ± 0.003 | 0.048 ± 0.003 | 0.044 ± 0.003 | 0.043 ± 0.006 |
| benzaldehyde | 0.117 ± 0.002 | 0.131 ± 0.003 | 0.115 ± 0.002 | 0.113 ± 0.029 |
| cysteine | 0.020 ± 0.003 | 0.023 ± 0.001 | 0.020 ± 0.002 | 0.019 ± 0.002 |
| ethanol | 0.295 ± 0.015 | 0.129 ± 0.003 | 0.293 ± 0.006 | 0.126 ± 0.002 |
| ethanolamine | 0.026 ± 0.007 | 0.025 ± 0.004 | 0.029 ± 0.005 | 0.036 ± 0.003 |
| fumarate | 0.289 ± 0.001 | 0.106 ± 0.003 | 0.281 ± 0.003 | 0.107 ± 0.002 |
| galactose | 0.778 ± 0.021 | 0.728 ± 0.017 | 0.724 ± 0.006 | 0.702 ± 0.020 |
| glutamate | 0.526 ± 0.003 | 0.512 ± 0.007 | 0.525 ± 0.005 | 0.509 ± 0.012 |
| glucose | 1.98 ± 0.08 | 1.85 ± 0.09 | 1.82 ± 0.12 | 2.1 ± 0.10 |
| gluconate | 0.661 ± 0.005 | 0.639 ± 0.010 | 0.630 ± 0.023 | 0.600 ± 0.008 |
| glucouronate | 0.922 ± 0.009 | 0.954 ± 0.071 | 0.939 ± 0.027 | 0.984 ± 0.025 |
| glutamine | 0.436 ± 0.021 | 0.433 ± 0.023 | 0.384 ± 0.057 | 0.440 ± 0.012 |
| glycine | 0.060 ± 0.005 | 0.092 ± 0.009 | 0.074 ± 0.005 | 0.065 ± 0.008 |
| histidine | 0.512 ± 0.017 | 0.475 ± 0.012 | 0.581 ± 0.030 | 0.530 ± 0.026 |
| isoleucine | 0.122 ± 0.008 | 0.123 ± 0.006 | 0.131 ± 0.009 | 0.146 ± 0.001 |
| α-ketoglutarate | 0.337 ± 0.005 | 0.121 ± 0.002 | 0.320 ± 0.012 | 0.126 ± 0.004 |
| leucine | 0.133 ± 0.10 | 0.128 ± 0.006 | 0.147 ± 0.013 | 0.153 ± 0.001 |
| lysine | 0.097 ± 0.001 | 0.100 ± 0.001 | 0.103 ± 0.007 | 0.110 ± 0.003 |
| malate | 0.328 ± 0.001 | 0.122 ± 0.004 | 0.321 ± 0.006 | 0.122 ± 0.002 |
| malonate | 0.105 ± 0.005 | 0.100 ± 0.003 | 0.102 ± 0.001 | 0.104 ± 0.005 |
| methionine | 0.096 ± 0.013 | 0.076 ± 0.006 | 0.097 ± 0.006 | 0.080 ± 0.002 |
| octanoate | 0.017 ± 0.002 | 0.019 ± 0.003 | 0.020 ± 0.004 | 0.017 ± 0.004 |
| ornithine | 0.162 ± 0.010 | 0.154 ± 0.003 | 0.149 ± 0.006 | 0.166 ± 0.012 |
| pectin | 0.578 ± 0.017 | 0.597 ± 0.021 | 0.564 ± 0.024 | 0.564 ± 0.003 |
| phenol | 0.029 ± 0.002 | 0.030 ± 0.005 | 0.029 ± 0.022 | 0.032 ± 0.001 |
| phenylalanine | 0.122 ± 0.012 | 0.107 ± 0.011 | 0.132 ± 0.011 | 0.108 ± 0.006 |
| proline | 0.389 ± 0.047 | 0.400 ± 0.020 | 0.423 ± 0.015 | 0.399 ± 0.039 |
| propionate | 0.584 ± 0.005 | 0.113 ± 0.002 | 0.528 ± 0.018 | 0.115 ± 0.002 |
| serine | 0.213 ± 0.004 | 0.215 ± 0.020 | 0.234 ± 0.003 | 0.227 ± 0.006 |
| sorbitol | 0.406 ± 0.004 | 0.376 ± 0.018 | 0.404 ± 0.033 | 0.356 ± 0.014 |
| succinate | 0.285 ± 0.015 | 0.116 ± 0.003 | 0.272 ± 0.015 | 0.118 ± 0.003 |
| sucrose | 0.304 ± 0.011 | 0.283 ± 0.015 | 0.313 ± 0.005 | 0.275 ± 0.004 |
| taurine | 0.110 ± 0.007 | 0.110 ± 0.003 | 0.103 ± 0.002 | 0.114 ± 0.003 |
| tryptophan | 0.253 ± 0.013 | 0.250 ± 0.021 | 0.242 ± 0.027 | 0.252 ± 0.039 |
| threonine | 0.304 ± 0.024 | 0.259 ± 0.006 | 0.317 ± 0.022 | 0.289 ± 0.006 |
| valine | 0.086 ± 0.003 | 0.090 ± 0.009 | 0.078 ± 0.005 | 0.110 ± 0.001 |
| xylose | 0.098 ± 0.001 | 0.100 ± 0.001 | 0.095 ± 0.001 | 0.095 ± 0.001 |
|  |  |  |  |  |
| **Nitrogen** |  |  |  |  |
| acetamide | 0.152 ± 0.006 | 0.148 ± 0.013 | 0.156 ± 0.012 | 0.150 ± 0.035 |
| alanine | 1.77 ± 0.12 | 1.24 ± 0.01 | 1.69 ± 0.02 | 1.05 ± 0.08 |
| ammonium | 1.42 ± 0.03 | 1.31 ± 0.05 | 1.30 ± 0.03 | 1.61 ± 0.02 |
| arginine | 0.233 ± 0.008 | 0.223 ± 0.011 | 0.235 ± 0.007 | 0.216 ± 0.006 |
| asparagine | 1.59 ± 0.02 | 1.53 ± 0.02 | 1.43 ± 0.01 | 1.25 ± 0.02 |
| aspartate | 2.06 ± 0.07 | 1.86 ± 0.05 | 1.92 ± 0.09 | 1.43 ± 0.01 |
| carnitine | 0.162 ± 0.004 | 0.139 ± 0.020 | 0.151 ± 0.002 | 0.121 ± 0.005 |
| cysteine | 0.004 ± 0.001 | 0.002 ± 0.001 | 0.005 ± 0.001 | 0.003 ± 0.001 |
| ethanolamine | 0.031 ± 0.011 | 0.040 ± 0.004 | 0.030 ± 0.011 | 0.043 ± 0.005 |
| glutamate | 2.87 ± 0.05 | 2.91 ± 0.13 | 3.40 ± 0.03 | 2.90 ± 0.12 |
| glutamine | 1.31 ± 0.03 | 1.31 ± 0.01 | 1.46 ± 0.02 | 1.27 ± 0.01 |
| glycine | 0.049 ± 0.005 | 0.084 ± 0.003 | 0.054 ± 0.022 | 0.030 ± 0.002 |
| histidine | 3.06 ± 0.04 | 3.17 ± 0.02 | 3.33 ± 0.05 | 2.85 ± 0.01 |
| isoleucine | 0.265 ± 0.003 | 0.271 ± 0.013 | 0.196 ± 0.012 | 0.375 ± 0.176 |
| leucine | 0.267 ± 0.003 | 0.304 ± 0.007 | 0.222 ± 0.004 | 0.276 ± 0.020 |
| lysine | 0.135 ± 0.006 | 0.122 ± 0.002 | 0.135 ± 0.010 | 0.106 ± 0.007 |
| nitrate | 0.225 ± 0.003 | 0.132 ± 0.007 | 0.223 ± 0.007 | 0.121 ± 0.004 |
| nitrite | 0.085 ± 0.003 | 0.074 ± 0.001 | 0.095 ± 0.004 | 0.075 ± 0.001 |
| ornithine | 0.566 ± 0.003 | 0.217 ± 0.005 | 0.604 ± 0.030 | 0.207 ± 0.10 |
| phenylalanine | 0.220 ± 0.016 | 0.215 ± 0.014 | 0.199 ± 0.008 | 0.208 ± 0.004 |
| proline | 0.541 ± 0.20 | 0.249 ± 0.012 | 0.580 ± 0.017 | 0.246 ± 0.002 |
| serine | 1.72 ± 0.04 | 0.948 ± 0.018 | 1.89 ± 0.05 | 1.00 ± 0.01 |
| threonine | 0.435 ± 0.007 | 0.429 ± 0.051 | 0.371 ± 0.017 | 0.419 ± 0.008 |
| tryptophan | 0.021 ± 0.002 | 0.023 ± 0.001 | 0.026 ± 0.001 | 0.023 ± 0.001 |
| urea | 0.235 ± 0.024 | 0.291 ± 0.032 | 0.256 ± 0.048 | 0.235 ± 0.015 |
| valine | 0.231 ± 0.027 | 0.240 ± 0.006 | 0.249 ± 0.010 | 0.203 ± 0.003 |
| xanthine | 0.524 ± 0.027 | 0.186 ± 0.016 | 0.504 ± 0.027 | 0.174 ± 0.013 |
|  |  |  |  |  |
| **Sulfur** |  |  |  |  |
| cysteine | 0.160 ± 0.091 | 0.050 ± 0.034 | 0.101 ± 0.093 | 0.100 ± 0.090 |
| HEPES | 1.54 ± 0.05 | 1.42 ± 0.03 | 1.44 ± 0.05 | 1.37 ± 0.04 |
| methanesulfonate | 0.800 ± 0.045 | 0.808 ± 0.013 | 0.776 ± 0.024 | 0.800 ± 0.030 |
| methionine | 0.786 ± 0.042 | 0.726 ± 0.016 | 0.746 ± 0.045 | 0.688 ± 0.034 |
| MOPS | 1.47 ± 0.08 | 1.32 ± 0.08 | 1.35 ± 0.10 | 1.58 ± 0.09 |
| PIPES | 0.612 ± 0.031 | 0.610 ± 0.025 | 0.590 ± 0.009 | 0.884 ± 0.260 |
| sulfate | 1.98 ± 0.08 | 1.85 ± 0.09 | 1.82 ± 0.12 | 2.10 ± 0.10 |
| taurine | 0.389 ± 0.047 | 0.400 ± 0.019 | 0.423 ± 0.015 | 0.399 ± 0.039 |
